# Opinion review of drug resistant tuberculosis in West Africa: tackling the challenges for effective control

**DOI:** 10.3389/fpubh.2024.1374703

**Published:** 2024-05-16

**Authors:** Isaac Darko Otchere, Adwoa Asante-Poku, Kodjo Francis Akpadja, Awa Ba Diallo, Adama Sanou, Prince Asare, Stephen Osei-Wusu, Nneka Onyejepu, Bassirou Diarra, Yaotsè Anoumou Dagnra, Aderemi Kehinde, Martin Antonio, Dorothy Yeboah-Manu

**Affiliations:** ^1^Bacteriology Department, Noguchi Memorial Institute for Medical Research, University of Ghana, Accra, Ghana; ^2^Medical Research Council Unit The Gambia at the London School of Hygiene and Tropical Medicine, Banjul, Gambia; ^3^National Tuberculosis Reference Laboratory, Lome, Togo; ^4^Biological Sciences Department, Faculty of Pharmacy at Cheikh Anta Diop University, Dakar, Senegal; ^5^Centre Muraz, Institut National de Santé Publique, Bobo-Dioulasso, Burkina Faso; ^6^Unité de Formation et de Recherche en Sciences de la Vie et de la Terre, Université Nazi Boni, Bobo-Dioulasso, Burkina Faso; ^7^Microbiology Department, Center for Tuberculosis Research Laboratory, Nigerian Institute of Medical Research, Lagos, Nigeria; ^8^University Clinical Research Center, University of Sciences, Techniques and Technologies of Bamako, Bamako, Mali; ^9^Department of Medical Microbiology and Parasitology, College of Medicine, University of Ibadan, Ibadan, Nigeria; ^10^Department of Infection Biology, Faculty of Infectious and Tropical Diseases, London School of Hygiene and Tropical Medicine, London, United Kingdom; ^11^Centre for Epidemic Preparedness and Response, London School of Hygiene and Tropical Medicine, London, United Kingdom

**Keywords:** drug resistance, tuberculosis, West Africa, challenges, control measures, collaboration, WANETAM

## Abstract

Drug-resistant (DR) tuberculosis (TB) is a major public health concern globally, complicating TB control and management efforts. West Africa has historically faced difficulty in combating DR-TB due to limited diagnostic skills, insufficient access to excellent healthcare, and ineffective healthcare systems. This has aided in the emergence and dissemination of DR *Mycobacterium tuberculosis* complex (MTBC) strains in the region. In the past, DR-TB patients faced insufficient resources, fragmented efforts, and suboptimal treatment outcomes. However, current efforts to combat DR-TB in the region are promising. These efforts include strengthening diagnostic capacities, improving access to quality healthcare services, and implementing evidence-based treatment regimens for DR-TB. Additionally, many West African National TB control programs are collaborating with international partners to scale up laboratory infrastructure, enhance surveillance systems, and promote infection control measures. Moreso, novel TB drugs and regimens, such as bedaquiline and delamanid, are being introduced to improve treatment outcomes for DR-TB cases. Despite these obstacles, there is optimism for the future of DR-TB control in West Africa. Investments are being made to improve healthcare systems, expand laboratory capacity, and support TB research and innovation. West African institutions are now supporting knowledge sharing, capacity building, and resource mobilization through collaborative initiatives such as the West African Network for TB, AIDS, and Malaria (WANETAM), the West African Health Organization (WAHO), and other regional or global partners. These efforts hold promise for improved diagnostics, optimized treatment regimens, and provide better patient outcomes in the future where drug-resistant TB in WA can be effectively controlled, reducing the burden of the disease, and improving the health outcomes of affected individuals.

## 1 Introduction

Tuberculosis (TB) caused by drug resistant (DR) strains of the *Mycobacterium tuberculosis complex* (MTBC) is called DR-TB whose treatment requires the use of relatively more toxic drugs for extended period of time with comparatively low success rate (1–4). Over the years, TB has been the leading cause of mortality by a single infectious disease until the recent COVID-19 pandemic. In 2022, the World Health Organization (WHO) reported 10.6 million new cases of TB and 1.6 million deaths (5). There was an estimated 410,000 cases of people who developed TB caused by MTBC that is resistant to rifampicin; the backbone of the Directly Observed Treatment Short-Course (DOTS) regimen (5). Africa, home to about 15.19% of humans on earth carries ~24% of the global TB burden (5). Unlike other geographical areas which harbor single to few lineages of the MTBC, WA harbors all the six major lineages of the MTBC (6, 7).

The primary risk factors for DR-TB infection include close contact with a DR-TB patient, history of TB treatment, cavity pulmonary TB, poor drug quality, inadequate supply of anti-TB drugs, treatment failure, poor adherence to treatment, inappropriate use of TB drugs and having a weakened immunity, such as in the case of HIV/AIDS, uncontrolled diabetes mellitus (DM), or malnutrition (8–12). Close contact with a DR-TB patients exposes one to potential inhalation of air droplets containing DR-TB bacilli released by infected patient. Therefore, if such a person develops TB from this exposure, it is very likely that it will be DR-TB from the onset. History of TB treatment presupposes that a person had a prior exposure to anti-TB drugs. However, among TB patients with multiple episodes of TB, molecular characterization has shown that there is more relapse than reinfection (13, 14). Exposure to anti-TB drugs can serve as selective pressure for DR-MTBC (15, 16). Therefore, TB patients with history of treatment are more likely to be DR-TB patients. On the other hand, poor adherence to treatment, inadequate supply of drugs and poor drug quality can lead to suboptimal concentration of drugs in the blood stream of infected patients. Thus, promoting selection of DR-MTBC and development of DR-TB during treatment (17–19). Again, improper prescription practices, such as using suboptimal drug regimens or using the wrong combination of drugs, can contribute to the development of drug resistance. For example, misuse of TB drugs in the treatment of other infections can contribute to the emergence of DR-MTBC strains. Thence, the need to keep drugs at desired temperature, prescribe good quality medications, follow the prescribed treatment regimen for the full duration even if symptoms improve, and reserve TB drugs for treatment of TB disease only. Lastly, factors that can potentially compromise the immunity, including HIV/AIDs, uncontrolled DM and malnutrition, negatively impact recovery from TB infection by prolonging the time it takes to clear the bacilli thus, allowing the bacteria enough time to evolve to tolerate the drugs potentially leading to resistance (13–15, 17–20). Addressing these risk factors is crucial for preventing the emergence and spread of DR-TB and improving treatment outcomes.

Unlike other bacteria that can easily acquire DR-conferring genes through horizontal gene transfer, DR among the MTBC is mostly caused by point mutations within DR- associated genes or promotor region of such genes (21). Nevertheless, the emergence and fixation of mutations that cause resistance to specific drugs is influenced by the genetic background of the infecting MTBC (22–24). This shows that resistance to same drug can be caused by different mutations with respect to different lineages of the MTBC (25, 26). This could lead to misdiagnosis of DR-TB in West Africa (WA) which has unique lineages of the MTBC when using commercially available rapid diagnostic assays. There is, therefore, the need for research into studying the determinants of DR in WA to support global efforts to develop rapid TB diagnostics. This opinion review explores the various aspects of DR-TB, including its definitions, causes, prevalence, challenges in diagnosis and treatment, and the implications for public health in WA.

## 2 Types of drug-resistant tuberculosis

There are several categories of DR-TB based on the drugs to which the infecting bacteria have developed resistance (Table 1). Mono-resistance refers to resistance to any single TB drug. Multidrug-Resistant (MDR) TB (MDR-TB) is defined as resistance to at least isoniazid and rifampicin. These two drugs are the backbone of the first-line TB treatment regimen. They are critical for TB treatment because resistance to both drugs automatically leads to treatment failure with the first line regimen and significantly limits the available treatment options (27–29). Extensively DR-TB (XDR-TB) is a more severe form of DR-TB caused by an MDR-MTBC that has developed further resistance to any fluoroquinolone and at least one of bedaquiline and linezolid (30). This further narrows down the treatment options, making XDR-TB challenging to treat successfully (27). The precursor to XDR-TB is pre-XDR-TB which is any MDR-TB with additional resistance to any fluoroquinolone whereas Polyresistant (PR) TB is defined as resistance to at least two TB drugs excluding rifampicin (30).

**Table 1:** Description of categories of DR-TB.

**DR category**	**Description**
RR	Resistance to RIF
IR	Resistance to INH
MDR	Resistance to RIF & INH
pre-XDR	MDR with additional resistance to a FQ
XDR	MDR with additional resistance to an FQ and at least one of LZD and BDQ
PR	Resistance to at least two drugs excluding RIF

## 3 Prevalence and distribution of drug-resistant tuberculosis in West Africa

**Figure 1:**
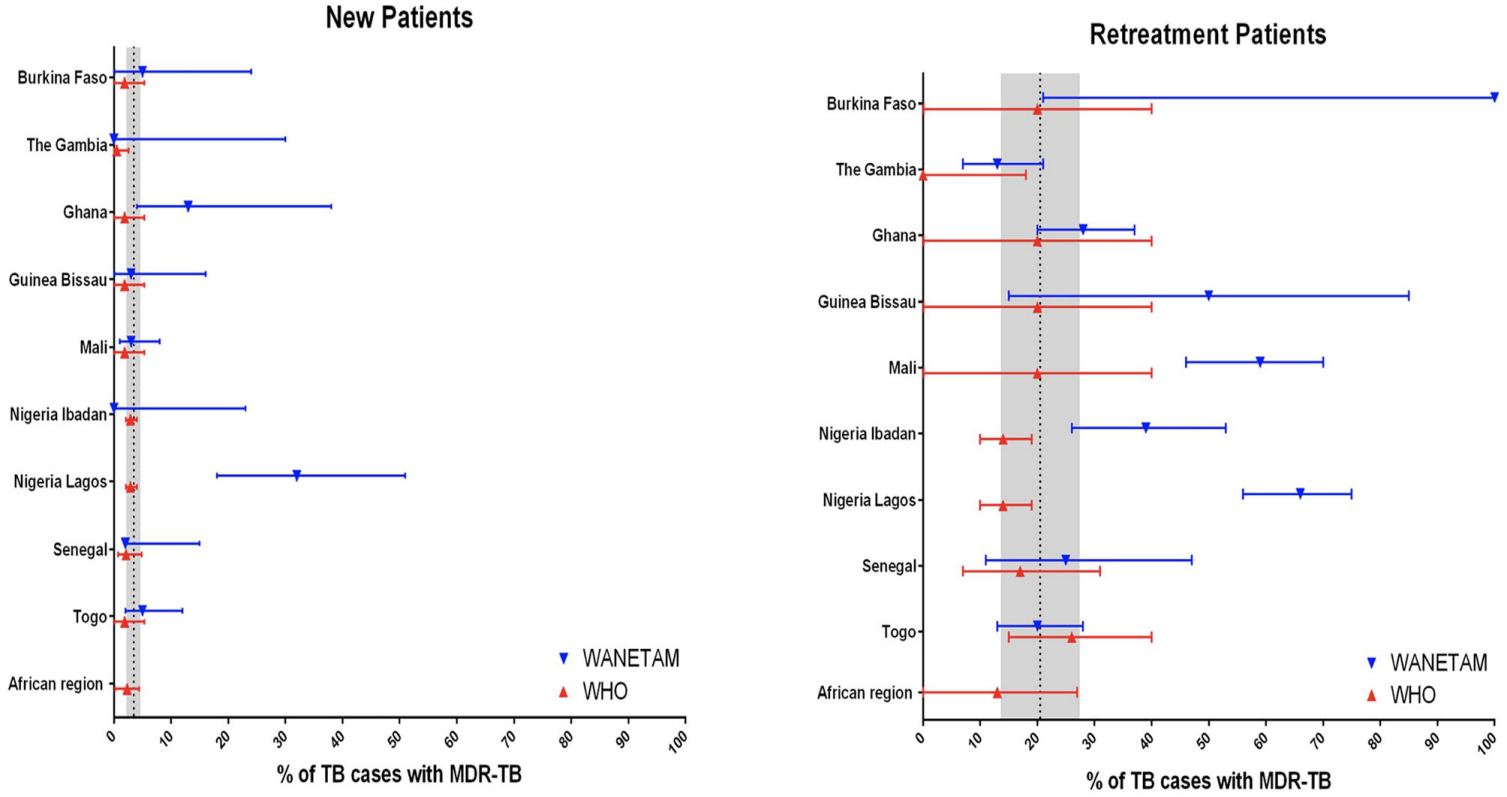
Comparing the 2016 WHO estimates of MDR-TB with actual incidence in nine West African countries [Adopted from Gehre et al. (33)].

**Figure 2:**
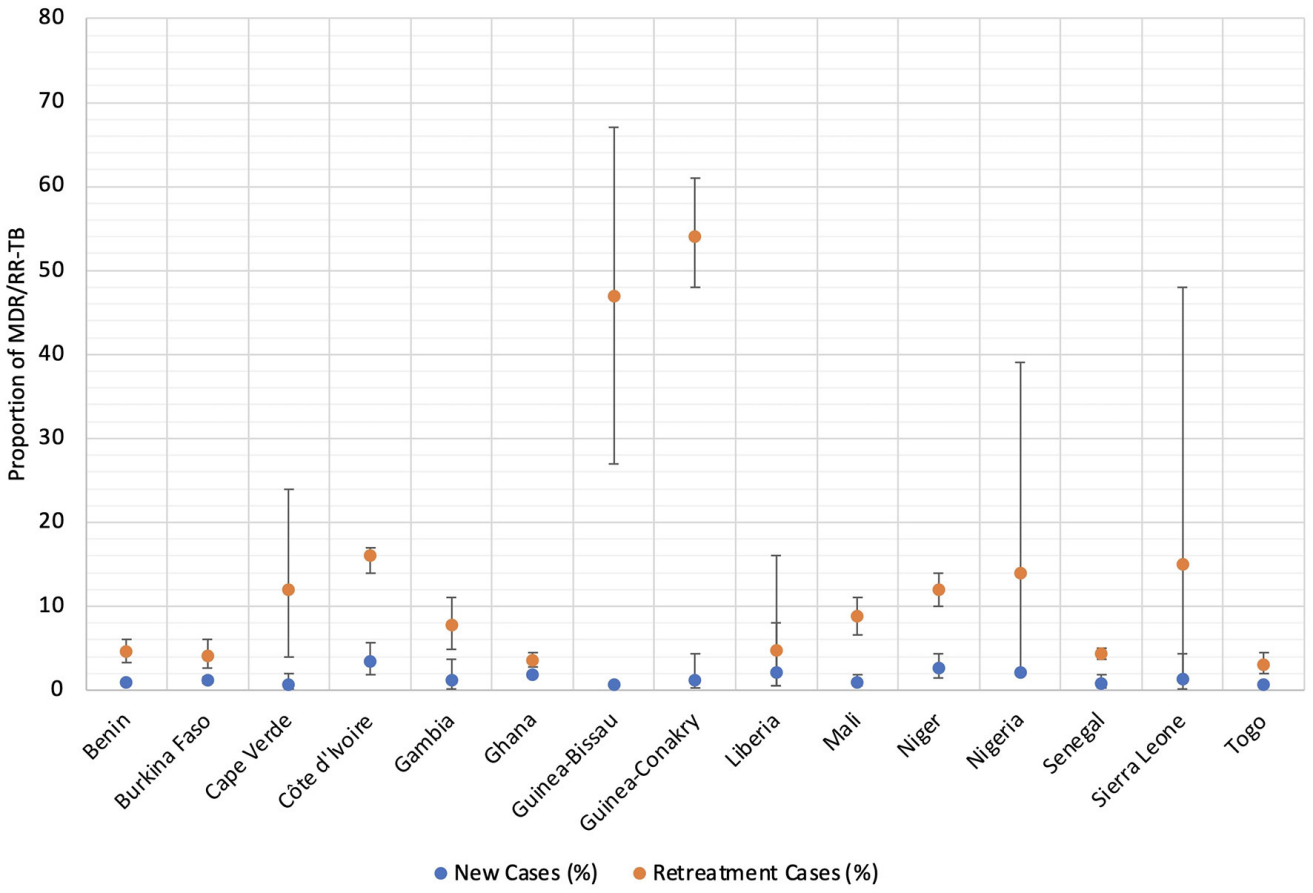
Comparing the 2022 WHO estimates of MDR/RR-TB between new and retreatment TB cases in West Africa (According to Global tuberculosis report, (5)).

Data on DR-TB in WA are scanty which reflect diagnostic limitation but not actual epidemiologic situation (31). Drug-resistant TB including but not limited to MDR and XDR is an emerging public health problem in WA with several countries reporting different prevalence over the years (7, 32–35). A collaborative DR-TB surveillance among nine WA countries by the West African Network for Tuberculosis, AIDS and Malaria (WANETAM) showed that the WHO estimates are almost always below the actual incidence (Figure 1) (33). While the WHO estimated the prevalence of MDR-TB in new and retreatment cases to be 2 and 17%, respectively, this study which analyzed 974 bacterial isolates found 6 and 35%, respectively. It was found that 39% of isolates (corresponding to 39% of TB patients) were resistant to at least one first-line TB drug whereas 22% were MDR/RR-TB. Of concern is that, pre-XDR-TB cases were found in all the eight participating countries (33). The most recent WHO TB report shows different burden of MDR/RR-TB across WA ranging from least 0.67% among new TB cases in Togo to the highest 54% among previously treated TB cases in Guinea-Conakry as summarized below (Figure 2). However, getting the actual current prevalence and distribution of DR-TB in WA is essential for effective control and future management strategies. This section reviews the current burden and distribution of DR-TB in WA.

### 3.1 Benin

The WHO estimated 0.93 and 4.6% MDR/RR-TB, respectively, among new and retreatment cases (5). However, there are varying prevalence rates of DR-TB reported in the country including 1.6 and 11% among new and previously treated TB patients, respectively (36), similar to another report of 12% MDR-TB among retreatment cases (34).

### 3.2 Burkina Faso

The WHO estimates of MDR/RR-TB in the country are 1.2 and 4.1% among new and retreatment cases, respectively (5), a slight reduction from 1.6 to 14% estimated in 2016. However, national survey between 2006 and 2007 found 2 and 14.5% MDR-TB, respectively, among new and retreatment cases (37).

### 3.3 Cape Verde

The island country has an estimated 0.69 and 12% MDR/RR-TB among new and previously treated TB cases, respectively, whereas the actual number of cases confirmed stood at two patients who were all put on an appropriate treatment regimen (5).

### 3.4 Côte d'Ivoire

The estimated figures are 3.5 and 16% MDR/RR-TB, respectively, among new and retreatment cases in the country (5). This correlates with a 10-year national survey which found 4.5 and 22% RR-TB among new and previously treated TB cases in the country (35). A cohort of 81 MDR-TB patients monitored in a pneumology unit, faced debilitating side effects leading to dropouts (39.2%), deaths (15.2%), diverse attacks (30.4%), and only 5% cured (38).

### 3.5 Gambia

The WHO estimated 1.2 and 7.8% MDR/RR-TB among new and retreatment cases, respectively (5). However, an earlier study reported no MDR-TB among new cases but 12.6% among retreatment cases in The Country (33).

### 3.6 Ghana

The WHO estimated 1.9 and 3.6% MDR/RR-TB among new and retreatment cases in Ghana (5). The first national DR-TB survey reported MDR/RR-TB rate of 3.1% (39) whereas a recent survey also reported a high rate of 5.6% (40) with 3.1% among new cases and as high as 33.3% among retreatment cases. Other studies in different parts of the country reported MDR/RR rates of 2.9% in the Greater Accra region (41), 3.31% in the Southern and Northern sectors (23), 3.5% in the Southern and Northern sectors (42), 3.1% in the Northern sector (43), and 8.1% in the Volta region (44). Additionally, a recent analysis of MTBC isolates from difficult-to-treat TB cases from Ghana over 2 years reported MDR/RR rate of 42% and pre-XDR-TB rate of 6.4% (45) agreeing with an earlier reported higher proportion of pre-XDR-TB (35%) among MDR-TB patients in the first pre-XDR-TB (33, 46) and XDR-TB case in the country (47).

### 3.7 Guinea-Bissau

The WHO estimated 0.73 and 47% MDR/RR-TB, respectively, among new and retreatment TB cases in the country (5). A study in the country also estimated 10 and 40% MDR/RR-TB, respectively, among new and retreatment cases but a mere 25% of these cases are diagnosed (48). To exacerbate the situation, about 45% of the diagnosed never complete treatment and for the remaining 55% who completed the treatment, success rate was only 34% (48).

### 3.8 Guinea-Conakry

The WHO estimated 1.2 and 54% MDR/RR-TB among new and retreatment cases, respectively (5). However, there is limited information on the actual burden of TB as well as DR-TB in the country. Nevertheless, there is a report of a high treatment cure rate (80.6%) MDR-TB in the country (49).

### 3.9 Liberia

As one of the three high TB burden countries in WA, the WHO estimated 2.1 and 4.8% MDR/RR-TB among new and retreatment cases, respectively (5). However, report by Borgenproject.org in collaboration with the national TB control program stipulates 2.5% prevalence of MDR/RR-TB in the country (https://borgenproject.org/tuberculosis-in-liberia/). Contrary to these estimates, two independent studies have reported very high MDR-TB prevalence of including 38.3% (50) and 23.5% (51) in the country.

### 3.10 Mali

The WHO estimate 1 and 8.8% MDR/RR-TB (5) contrary to the 3.3 and 58.6% MDR-TB (33), respectively, among new and retreatment TB cases in Mali. A similar report (3.4 and 66.3% among new and retreatment TB patients, respectively) has also been published (52). Furthermore, the investigators identified 18.8% resistance to second-line TB drugs among a random 38 (50%) of MDR isolates. The first 2 cases of XDR-TB were reported in 2017 with genomic evidence of nosocomial transmission from the first patient to the second. Of great concern, it took 10 months after diagnosis for the patients to receive appropriate treatment (53).

### 3.11 Niger

The WHO estimated 2.7 and 12% MDR/RR-TB among, respectively, new and previously treated TB cases in the country. The country seems to have a good program with 132 out of the 135 confirmed cases put on an appropriate treatment regimen (5).

### 3.12 Nigeria

Nigeria is one of the WHO recognized 30 high MDR/RR-TB burden countries with estimated 2.1% among new cases and 14% among previously treated TB cases (5). A recent study reported 3.5 and 7% MDR-TB among new cases and retreatment cases, respectively, in the country (54). However, relatively high prevalence of MDR/RR-TB 6 and 32%, respectively, among new and retreatment cases was also reported from a systematic review of studies from different parts of the country (55). A study did not distinguish new and retreatment cases reported MDR/RR-TB rate of 6.3 and 0.8% pre-XDR-TB (56).

### 3.13 Senegal

The WHO estimated 0.82 and 4.4% MDR/RR-TB, respectively, among new and retreatment cases (5). The National TB Control Program (NTP) of Senegal reported an increase in number of MDR-TB cases from 36 in 2010 to 77 in 2015 and MDR-TB rates of 0.5 and 17.9% among new and previously treated TB cases in 2014. However, an independent study in Senegal found the rate of MDR-TB to be 5.5 and 25% among new cases and retreatment cases, respectively (33).

### 3.14 Sierra Leone

There has been no national survey but the WHO estimated 1.3 and 15% MDR/RR-TB among new and retreatment cases, respectively (5). A recent study reported 14.7% MDR/RR-TB incidence (57) and a systematic review of reports from the country showed that among 628 identified MDR/RR-TB cases, 440 (70.1%) were retreatment cases with most being males (374/440) (58).

### 3.15 Togo

The WHO estimated 0.67 and 3% MDR/RR-TB, respectively, among new and retreatment cases (5). Nevertheless, a study monitoring a cohort of previously treated TB patients using GeneXpert and BACTEC MGIT found 24 and 25% of MDR-TB cases, respectively (59).

## 4 Challenges in diagnosis and treatment of drug-resistant tuberculosis

### 4.1 Diagnostic methods for detecting DR-TB and challenges

Accurate and timely diagnosis of DR-TB is essential for appropriate treatment plan. Several diagnostic methods including both phenotypic (culture-based) and DNA-based tests are available for detecting DR-TB. Due to the drawbacks of culture-based drug susceptibility testing (DST) methods such as requiring specialized laboratories, high expertise and often take weeks to provide results (60, 61), DNA-based using of polymerase chain reaction (PCR) are preferably used. For example, many of the countries are usingXpert MTB/RIF assay (Cepheid) detects rifampicin resistance (RR) and the line probe assays (Hain LifeSciences) (62). Nevertheless, these may not be accessible in some remote settings (63, 64). Furthermore, difficulty in maintenance of these equipment leads to referral of diagnostic services to distant reference labs leading to delayed diagnosis and initiation of appropriate treatment (64–66). Additionally, the paucibacillary nature of TB among HIV co-infected cases and children poses a diagnostic challenge, resulting in false-negative results and eventual underestimation of DR-TB (67).

### 4.2 Drug resistant TB treatment challenges

Treating DR-TB requires the use of multiple drugs for an extended duration, typically from 6 to 9 months (https://www.cdc.gov/tb/topic/drtb/bpal/default.htm#print). This long and complex treatment regimen poses significant challenges. First, the combination of drugs used can have significant side effects, including gastrointestinal disturbances, hepatotoxicity, ototoxicity, and psychiatric effects (68). These side effects can potentially lead to treatment discontinuation, non-adherence, and hence decreased treatment effectiveness. Secondly, availability of these second-line drugs may be limited in resource-constrained settings such as WA, affecting the ability to provide appropriate treatment to all diagnosed DR-TB patients (69). For example, out of the total 1,131 MDR-TB cases diagnosed in Ghana from 2018 to 2022, those that were put on treatment were 956 (84.5%) (Ghana, National Tuberculosis Control Program, Annual Report 2022, Unpublished). Albeit this proportion can be considered one of the highest in WA, it is still below the 90% enrolment recommended by the WHO (70). Consequently, limited access to these drugs can potentially lead to suboptimal treatment regimens that compromise treatment outcomes and contribute to the development of additional drug resistance.

## 5 Public health challenges for control of DR-TB in West Africa

Preventing the emergence and spread of DR-TB in WA is a multifaceted problem that demands a wholistic solution. Firstly, limited access to rapid diagnostics for accurate and timely detection of DR-TB leads to delayed diagnosis and inadequate treatment regimens (69, 71, 72). Delayed diagnosis compromises patient outcomes and allows for prolonged infectious period of DR-TB patients which potentiates transmission to others (73). Timely diagnosis and initiation of appropriate treatment are therefore crucial for better treatment outcomes that interrupt the transmission chain. Secondly, TB is highly stigmatized as a disease associated with either death or curse among many WA ethnic groups (64, 74, 75), thus the affected do not accept and seek help early, leading to high community transmissions (76).

Furthermore, due to the limited counseling at diagnosis and community engagement, some TB patients after diagnosis spend prolonged periods seeking spiritual assistance. Some also alternates between taking certified TB drugs from the clinics and seeking spiritual help leading to emergence and spread of DR-TB and poor treatment outcomes (77–79). Thus, incompetent treatment and poor adherence to treatment regimen undermines positive treatment outcomes (80, 81). Additionally, difficulty in tracing contacts of DR-TB patients for screening also contribute to delay in identifying potentially infected DR-TB patients for proper management (82). Contact tracing can further be complicated by the inability of sputum-based diagnostics to pick-up latent infections which may progress to active disease later (82–84).

Congregate settings, such as correctional facilities, homeless shelters, and refugee camps, create environments conducive for DR-TB spread (70). WA has over the past decade faced political instability in some countries leading to creation of several overcrowded refugee or internally displaced people camps with poor ventilation, and limited access to proper healthcare services conducive for spread of TB including DR-TB (85). Additionally, malnutrition and poor immunity negatively affect effectiveness of TB treatment and can lead to emergence of DR-TB (70). The interaction between TB and HIV can lead to more severe disease and treatment complications. The co-management of TB and HIV is a complex process involving multifaceted drug therapy, needing careful monitoring of drug-drug interactions (86). Therefore, the high prevalence of HIV/AIDS in WA further exacerbates the challenge of managing DR-TB. Notwithstanding these factors, the contribution of the TB bacteria itself to emergence, evolution and spread of DR cannot be overlooked. WA is a hot-spot of MTBC diversity with seven of the nine phylogenetic lineages including two WA restricted lineages with some genotypes associated with DR as well as specific DR-conferring and/or associated mutations (7, 23, 87–91).

Drug resistant MTBC strains require more expensive and prolonged treatment regimens, straining healthcare systems and diverting resources from other public health priorities (70, 71, 92, 93). This is even more apparent in resource limited settings in WA where the demand for these resources overwhelms supply. The high cost and intermittent supply of diagnostics and medications for DR-TB limits their accessibility and subsequently hinder effective control and management of DR-TB (71). DR-TB undermines TB control efforts by reducing treatment success rate and increasing cost of management (94). The average treatment success rate for uncomplicated TB is 85% whereas that for MDR/RR-TB albeit more expensive is reduced to 60% and that for pre-XDR/XDR-TB is even worse and requires using relatively more expensive drugs compared to MDR-TB (70, 95). With the continuous increase in the number of drugs becoming ineffective against the MTBC, the affected TB patients are gradually challenged with limited treatment options.

The Economic Community of West African States (ECOWAS) allows free movement of goods and services among member states. While there is daily heavy movement of people including nomads, herdsmen and refugees as well as animals along approved and unapproved routes (96–98), the sub-region has no border control measures. The non-existent TB testing strategies at these ports of entry concomitant with TB endemicity of the region, encourages people and/or livestock with either active or latent TB infection (LTBI) including DR-TB to cross borders unhindered with it accompanying public health implications (36, 99). There is therefore the need for regional collaborations and coordination in addressing DR-TB through regional active surveillance and creation of awareness.

## 6 Potential consequences of uncontrolled DR-TB

The consequences of uncontrolled DR-TB transmission are far-reaching. Firstly, it will result in hyper transmission of DR-TB and consequently a higher DR-TB burden and increased mortality (100, 101). Again, uncontrolled DR-TB can result in economic burdens at both individual and societal levels, because the costs of extended treatment regimens, hospitalization, and the loss of productivity have profound implications for individuals and healthcare systems (102–104). The economic impact can be exacerbated because DR-TB disproportionately affects poor populations, thus furthering poverty and inequality (105–107). Moreover, uncontrolled transmission of DR-TB in WA will compromise global health security because the interconnectedness of countries and ease of travel within the sub-region and overseas will facilitate the spread of DR-TB across borders, posing a threat to populations worldwide (70, 86).

## 7 Strategies for addressing drug-resistant tuberculosis in West Africa

### 7.1 Strengthening healthcare infrastructure and capacity

A critical step in addressing DR-TB in WA is political will of respective governments to reduce overreliance on foreign aid (71, 108). They must be committed to strengthen their health care systems to be more responsive/sensitive and resilient. Notwithstanding the significant help these aid agencies offer, their activities are affected by shocks such as outbreaks of other diseases as was seen with COVID-19 (64, 66, 86, 109, 110). Therefore, ensuring uninterrupted DR-TB control activities in WA demands governments in WA to set aside significant portions of their annual budgets toward improving laboratory facilities for accurate and timely diagnosis of DR-TB, ensuring access to DST, and establishing robust treatment programs that do not run out of required drugs (71, 111). In addition to physical structure and supplies, there is also the need to offer specialized training for healthcare providers in the diagnosis, management, and treatment of DR-TB, as well as improving infection control (112, 113).

### 7.2 Enhanced surveillance for early detection of DR-TB

There is the need for comprehensive surveillance system that tracks DR-TB patterns and monitors the spread of resistant strains in endemic communities (82, 114). Such surveillance system requires sensitive, rapid diagnostics for point-of-care testing and real-time data collection analysis tools to promote early detection of outbreaks as well as monitor cross-border importation/exportation of DR-TB (115). The deployment of GeneXpert MTB/RIF in WA, allowing dual diagnosis of TB and rifampicin resistance within hours is commendable (116, 117). The new GeneXpert MTB/XDR deployed in few reference labs in the sub-region work in concert with the GeneXpert MTB/RIF to give better resolution on MTBC susceptibility to RIF, INH, RQs and (118, 119). Nevertheless, the GeneXpert is inapplicable as a point of care tool in rural areas. On the other hand, the newly introduced Seegene-Anyplex-II-MTB/MDR/XDR (120), a real-time PCR-based diagnostics may account for some limitations of the GeneXpert platform, but requires evaluation in WA the hot-spot of MTBC diversity to access its sensitivity and specificity (7, 121, 122). Furthermore, efficient contact tracing and follow-up of individuals exposed to DR-TB patients with regular testing and screening can promote early detection of potential emerging cases and help prevent secondary transmissions whilst improving treatment outcomes with appropriate treatment regimens (123, 124).

### 7.3 Research into the impact of MTBC diversity on TB control

In the past two decade, some West African scientists are gradually making progress in basic scientific research toward understanding the biology of the MTBC (122, 125–130) and the potential impact of MTBC diversity on TB control (47, 131–133). This is because WA harbors seven out of the nine phylogenetic lineages (L1-L4, L7 & L8 making up *M. tuberculosis sensu stricto* [Mtbss] and L5, L6 & L9 making up *M. africanum* [Maf]) of the MTBC (6, 90) with a couple of WA-restricted several lineages (Maf lineages) and sub-lineages (Ghana and Cameroun sub-lineages of L4) (7). Some highlights of research findings from WA are described below. Maf transmits equally as Mtbss but progresses relatively slowly to active disease and is associated with relatively smaller transmission clusters (134, 135). Whereas, INH-resistance among Mtbss is driven by *katG* mutations conferring high-level resistance, INH-resistance among Maf is driven by *inhApro* mutations conferring low-level resistance (23). Maf lineages L5 and L6 are two distinct pathogens restricted to WA by unknown mechanisms (122). Maf L5 is also more likely to be associated with DR-TB compared to L6 similar to the association of Mtbss L4 Ghana genotype with DR-TB in WA (23, 43). Finally slower *in vitro* growth of Maf (136) in concomitance with its slower sputum-smear conversion rate (137) may warrant review of DOTS in West Africa as the 6 months may not be adequate for TB caused by Maf (138). Nevertheless, there is still room for further TB research in WA to contribute knowledge toward development of more efficient control tools.

### 7.4 Development of new drugs from traditional medicinal plants

The continuous evolution of DR limits treatment options for DR-TB and threatens to eventually make TB untreatable. Therefore, new and more effective drugs are crucial for combating DR-TB. Current treatment regimens for DR-TB are lengthy, complex, and associated with significant side effects (70). Investing in research and development of potent but less toxic drugs is essential whereas those that can give shorter treatment duration would be an added advantage. Most current efforts toward development of new TB treatment regimens involve exploring new drug targets and repurposing existing drugs (94, 139, 140). However, WA is home to numerous plant-based medicines for treating myriad of diseases including TB for centuries. Some of these plant preparations have recently been scientifically assessed for their anti-mycobacteria activity with promising outcomes (141). There is the need to invest in taking these promising preparations into pre-clinical trials for further development and advance research into identify and characterizing the active compound (s) as leads for new anti-TB drugs. Expedite development of novel anti-TB agents originating from the sub-region as well as integration of traditional, complementary, and alternative medicine into the health care system, particularly in rural areas where they are most prevalent, can enhance access to TB treatment, provided safety and efficacy standards are met. The provision of independent expert advice and support to countries in this regard by the WHO and African Centres for Disease Control are thence commendable (142).

### 7.5 Efficient public education and awareness campaigns for promoting knowledge, dispelling myths, and encouraging behavioral change

There are many innovative communication platforms that can be used for public education on DR-TB including mass media, social media, community meetings, workshops, and educational materials tailored to different populations using local languages to enhance the reach and impact of the message (71, 143–147). Such campaigns must engage communities, healthcare providers, policymakers, and other influential persons including but not limited to celebrities and local leaders in efforts to prevent the spread of DR-TB (143). The campaign should rely on experts to provide accurate and accessible information about TB, its causes, transmission, and preventive measures to help dispel misconceptions surrounding TB that negatively impact timely diagnosis and treatment (70, 147–150). Dispelling such myths will dispel stigma and discriminations against TB patients and fostering understanding, empathy, and acceptance as well as support for those affected. Secondly, such campaigns must raise awareness on antibiotic stewardship that promotes responsible use of antibiotics emphasizing the importance of completing prescribed courses of treatment and avoiding the use of antibiotics without prescription to help protect the potency of available TB drugs (151, 152). In addition, there should be a system for monitoring the campaign activities and evaluating the impact of the campaign on knowledge acquisition and attitudinal changes in relation to TB and DR-TB in the catchment communities as well as identify areas for improvement. Finally, such public education efforts must be sustained for longer periods to ensure that the intended knowledge becomes part and parcel of the target.

### 7.6 Fostering collaborations and partnerships among stakeholders

Addressing the complex challenge of DR-TB requires collaboration and partnerships among stakeholders including national TB control programs, government agencies, non-governmental organizations, healthcare providers, donors, religious bodies, traditional leaders, industry, and academia across the region. Such collaborative efforts can help mobilize resources, share expertise, coordinate activities, and advocate for enactment of new policies as well as modification of existing policies to fight DR-TB in the sub-region (153, 154). The initiative by the West African Network for Tuberculosis, AIDs, and Malaria (WANETAM) including 14 institutions in eight West African countries needs commendation. WANETAM is funded by the European and Development Countries Clinical Trials Partnership (EDCTP) to build capacity in standardized laboratory techniques essential for clinical trials and public health relevant diagnostics and research since 2008 (https://wanetam.org). Over the years the network has trained several clinicians, laboratory technicians, research administrators and research scientists as well as built both physical and electronic infrastructure in WA contributing to the fight against diseases of public health importance including DR-TB. There is, however, the need for several of such within sub-region collaborations funded by local governments for sustainability.

## 8 Conclusion

The growing burden of DR-TB in WA demands collective action and a unified approach to combat this daunting public health challenge. The strategies and interventions discussed in this article, including strengthening healthcare infrastructure, enhancing surveillance and infection control measures, developing innovative rapid, accurate and affordable diagnostics, developing new potent but less toxic drugs, ensuring access to care, and fostering partnerships and collaborations with academic institutions, pharmaceutical companies, and NGOs provide a roadmap for addressing DR-TB in the sub-region. However, the actual fight against DR-TB requires sustained commitment and innovations to overcome the existing barriers and improve patient outcomes. To effectively combat DR-TB in WA, it is imperative for West African governments, regional bodies, international organizations, healthcare providers, researchers, and affected communities and other interested stakeholders to work together to mobilize resources, advocate for policy changes, and ensure the implementation of comprehensive interventions in the sub-region. By implementing these comprehensive strategies against DR-TB, we can achieve a sub-region that is free from the threat of TB including DR-TB and ensuring the wellbeing of people living in WA and beyond.

## Author contributions

IO: Conceptualization, Formal analysis, Validation, Visualization, Writing—original draft, Writing—review & editing. AA-P: Writing—review & editing. KA: Writing—review & editing. AD: Writing—review & editing. AS: Writing—review & editing. PA: Visualization, Writing—review & editing. SO-W: Writing—review & editing. NO: Writing—review & editing. BD: Writing—review & editing. YD: Writing—review & editing. AK: Writing—review & editing. MA: Conceptualization, Data curation, Funding acquisition, Resources, Supervision, Validation, Writing—review & editing. DY-M: Conceptualization, Data curation, Funding acquisition, Resources, Supervision, Validation, Writing—review & editing.
